# Prognostic Value of KIF2A and HER2-Neu Overexpression in Patients With Epithelial Ovarian Cancer

**DOI:** 10.1097/MD.0000000000002803

**Published:** 2016-03-03

**Authors:** Di Wang, Huijun Zhu, Qing Ye, Chenyi Wang, Yunzhao Xu

**Affiliations:** From the Department of Obstetrics and Gynecology (DW, QY, CW, YX) and Department of Pathology (HZ), Nantong University Affiliated Hospital, Nantong, Jiangsu, China.

## Abstract

Kinesin family member 2A (KIF2A) is a member of Kinesin-13 family and involved in cell migration and cell signaling. Human epidermal growth factor receptor 2 (HER2-neu) is implicated in the development of many cancers. Both of these 2 proteins are upstream inducer of PI3K/AKT signaling pathway that plays an important role in the regulation of many cellular events including proliferation, survival, and invasion. We hypothesized that aberrant KIF2A and HER2-neu expression might be associated with aggressive behavior of epithelial ovarian cancer (EOC).

To address the prognostic implications of KIF2A and HER2-neu in EOC, we assessed protein levels of KIF2A and HER2-neu in 159 ovarian and fallopian tube tissues (111 carcinomas and 48 normal ovary or fallopian tube tissues) by immunohistochemistry (IHC) analysis on tissue microarray and KIF2A mRNA levels in 35 ovarian and fallopian tube tissues (15 carcinomas and 20 normal ovary or fallopian tube tissues) by real-time PCR.

We found that significantly higher KIF2A mRNA expression in EOC tumors than that in normal ovary or fallopian tube tissues. The IHC results showed that protein of KIF2A and HER2-neu was overexpressed in EOC tissues compared with normal ovary or fallopian tube tissues, and KIF2A expression level was significantly associated with lymph nodes, metastasis, ascites cells, and FIGO stage. No correlation between KIF2A and HER2-neu expression was observed. Survival analysis showed that patients with KIF2A and HER2-neu overexpression had a worse overall survival (OS) as compared to patients with low or none expression of the 2 proteins. Multivariate analysis of variance revealed that overexpression of KIF2A was an independent prognostic factor for OS.

These findings indicate the important role of KIF2A in predicting EOC prognosis.

## INTRODUCTION

Ovarian cancer is the deadliest gynecologic malignancies. Approximately more than 204,000 new ovarian cancer cases and 125,000 deaths occur yearly, constituting 4% of entire cancer cases and 4.2% of cancer deaths in women worldwide.^1^ Despite the fact that average survival time has been prolonged slightly over the past decades, 5-year survival rate for ovarian cancer remains under 50%.^2^ The poor prognosis is largely attributed to the poor understanding of the events that initiate ovarian cancer and promote the disease to progress. Biomarkers have been shown to play a critical role in the era of big data-driven personalized medicine, since they have greatly enhanced early cancer detection rate and help to ameliorate the clinical outcomes.^3–5^ Given the exceptional poor diagnosis of ovarian cancer, there is an urgent need to discover novel biomarkers with high sensitivity and specificity for the early diagnosis and targeted therapies of this disease.

Kinesin family member 2A (KIF2A) is a member of Kinesin-13 family. Human homolog of KIF2A is a motor protein engaged in transporting cargo proteins along microtubules, which specifically localizes to centrosomes during mitosis.^6^ It was reported that KIF2A silencing induced tumor cell apoptosis in vitro partially by suppressing the PI3K/AKT signaling pathway,^7^ a pathway commonly overactivated in a wide spectrum of human cancers. Another important protein-related PI3K/AKT pathway is human epidermal growth factor receptor 2 (HER2-neu, alias: *ERBB2*), which is implicated in carcinogenesis of various cancer types, including ovarian cancer.^8^ HER2-neu is a tyrosine kinase receptor in the epidermal growth factor family and plays an important role in cell proliferation and tumor cell metastasis. The PI3K/AKT pathway is a major downstream signaling pathway of HER2-neu.^9^ Amplification and overexpression of HER2-neu has been detected in up to 15% of breast cancers and in 7% to 20% of gastric cancers, which appeared to be a predictor of poor prognosis for cancer.^9–11^ Either gene amplification or overexpression may result in the dysregulation of HER2 signaling in epithelial ovarian cancer (EOC), thereby leading to excessive cell growth, DNA damage, and accelerating tumor progression.^12^ Taken together, these findings suggested that both KIF2A and HER2-neu might contribute to cancer progression, metastasis, and poor clinical outcomes partially through activating the PI3K/AKT signaling pathway.

So far, the relationship between KIF2A protein expression and clinical parameters in EOC has not been assessed. Moreover, prognostic values of combined KIF2A and HER2-neu remain unclear. Therefore, in this study, we aimed to determine expression levels of both HER2-neu and KIF2A protein in ovarian epithelial tumor samples and to analyze the correlation between KIF2A, HER2-neu, and other clinicopathological features in a cohort of EOC patients.

## MATERIALS AND METHODS

### Human Tissue Specimens and Patient Clinical Information

A total of 159 formalin-fixed paraffin-embedded tissue samples were included in this study, comprising 24 normal ovarian, 24 normal fallopian tube, and 111 EOC samples. EOC samples obtained from patients diagnosed with ovarian carcinoma who underwent initial surgery at the Gynecology Department of Nangtong University Affiliated Hospital from 2005 to 2009 were fixed in formalin and embedded in paraffin. The tissue samples with informed consent from patients were obtained at the time of surgery in the Department of Gynecology, Nantong University Affiliated Hospital. In order to achieve a maximal tumor resection, all ECO patients received standard operation and a platinum-based chemotherapy for at least 6 cycles after resection. None of the patients received any therapy against carcinoma prior to the surgical operation. Clinical information of the patients’ was recorded in detail, and the diagnoses were confirmed by at least 2 pathologists. Of the 111 ovarian cancer cases, 84 were serous carcinoma, and 27 cases were other types. There were 51 cases in stage I, 16 cases in stage II, 42 cases in stage III, and 2 cases in stage IV. With regard to histological grading, 90 cases were in high grade and 21 cases were in low grade. The average age of patients (24–78 years) was 51.26 ± 14.75 years. All patients were followed up from surgery through December 31, 2014. Besides, another 15 carcinoma samples and 20 normal ovary or fallopian tube tissue samples were collected for real-time PCR analysis. The present study was approved by the Ethics Committee of Nangtong University Affiliated Hospital.

### RNA Isolation and Quantification of Transcript Levels

The total RNA was isolated according to the protocol of TRIZOL reagent (Life Technologies). The LightCycler FastStart DNA Master SYBR Green I Kit (Roche Diagnostics, Tokyo, Japan) was used to carry out real-time PCR, following previously published procedure.^13^ Primers for KIF2A were as follows, forward primer 5′-GCCGAATACATCAAGCAAT-3′ and reverse primer 5′-CTCTCCAGGTCAATCTCTT-3′. Moreover, the housekeeping gene glyceraldehyde-3-phosphate dehydrogenase (GAPDH) gene was as follows, forward primer 5′-TGCACCACCAACTGCTTAGC-3′ and reverse primer 3′-GGCATGGACTGTGGTCATGAG-5′, to normalize KIF2A gene expression. Specific ways of reverse transcription and amplification were described by Fan et al.^14^

### Tissue Microarray Construction and Immunohistochemistry (IHC) Analysis

The experimental methods of tissue microarray construction in details were performed as previously described,^15^ by using tissue microarray system (Quick-Ray, UT06, UNITMA, Korea). KIF2A was detected by mouse monoclonal anti-KIF2A antibody (dilution 1:100) (Abcam, ab55383). HER2-neu was detected by rabbit anti-human HER2-neu antibody (dilution 1:50) (Dako, A0485). Not knowing about the clinical characteristics, 2 independent pathologists reviewed and scored the staining intensity of KIF2A and HER2-neu of each slide. Staining intensity, percentage of positive cells, and the product of the percentage and intensity score were described previously.^16^ We used the X-tile software program (The Rimm Lab at Yale University; http://www.tissuearray.org/rimmlab) to set the cutoff point that was statistically significant in terms of overall survival which described previously.^17^ For KIF2A and HER2-neu expression score, we used the cutoff 120 to assess as follows: 0 to 120: low expression and 121 to 300: high expression. For all subsequent analyses, KIF2A and HER2-neu protein expression levels were considered either as “Low” or “High” using these cutoff values.^16^

### Statistical Analysis

Statistical calculations of KIF2A mRNA expression were performed using *t* test. Chi-squared tests were used to evaluate whether KIF2A expression alone or combined with HER2-neu was correlated with clinicopathological factors of EOC. Patient survival curves were plotted using the Kaplan–Meier method. We adopted univariate and multivariate Cox regression models to evaluate prognostic significance. Data were analyzed using SPSS 20 statistics software (SPSS Inc., Chicago, IL) and STATA 12.0 (StataCorp, College Station, TX). For all analyses, we set *P* < 0.05 of statistical significance.

## RESULTS

### KIF2A mRNA Expression in EOC by PCR

**FIGURE 1:**
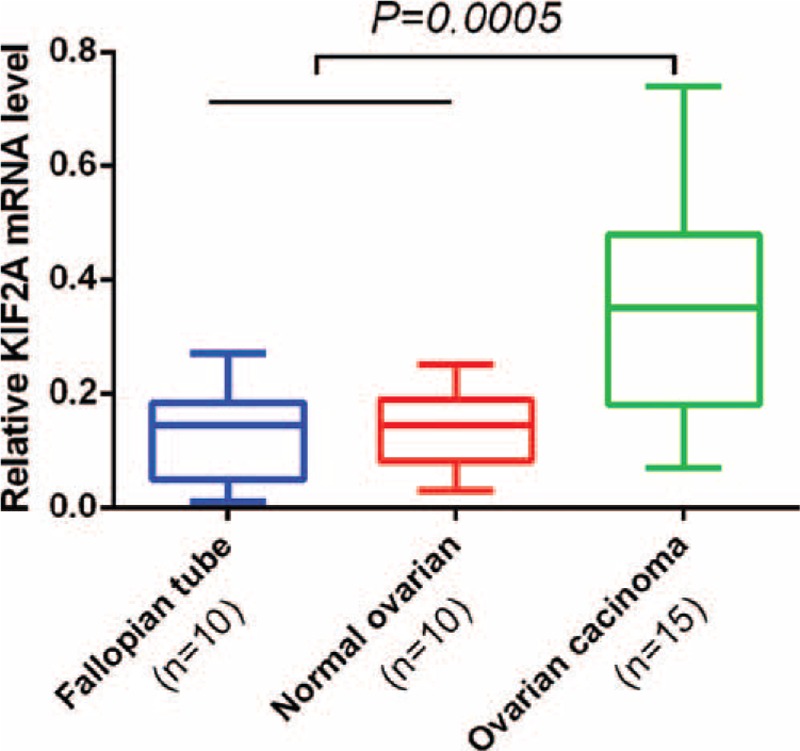
KIF2A mRNA expression in EOC tissues and normal tissues. Real-time PCR demonstrated that KIF2A mRNA expression in EOC, normal ovary, and normal fallopian tube tissues were 0.351 ± 0.203, 0.138 ± 0.068, and 0.125 ± 0.085, respectively. KIF2A mRNA expression in EOC samples was significantly higher than in normal ovarian tissue, fallopian tube tissue samples (all *P* < 0.05). EOC = epithelial ovarian cancer, KIF2A = Kinesin family member 2A.

To evaluate the KIF2A mRNA expression between EOC and normal tissue, RNA was isolated from 15 cases of ovarian carcinoma and 10 normal ovary and 10 normal fallopian tube tissue samples using real-time PCR. As is shown in Figure 1, the means of KIF2A mRNA in carcinoma and in noncancerous tissues (ovary and fallopian tube) were 0.351 ± 0.203, 0.138 ± 0.068, and 0.125 ± 0.085, respectively. PCR results showed higher expression of KIF2A mRNA in ovarian cancer samples than in noncancerous tissues (all *P* < 0.05).

### KIF2A and HER2-Neu Protein Expression in EOC by IHC

**FIGURE 2:**
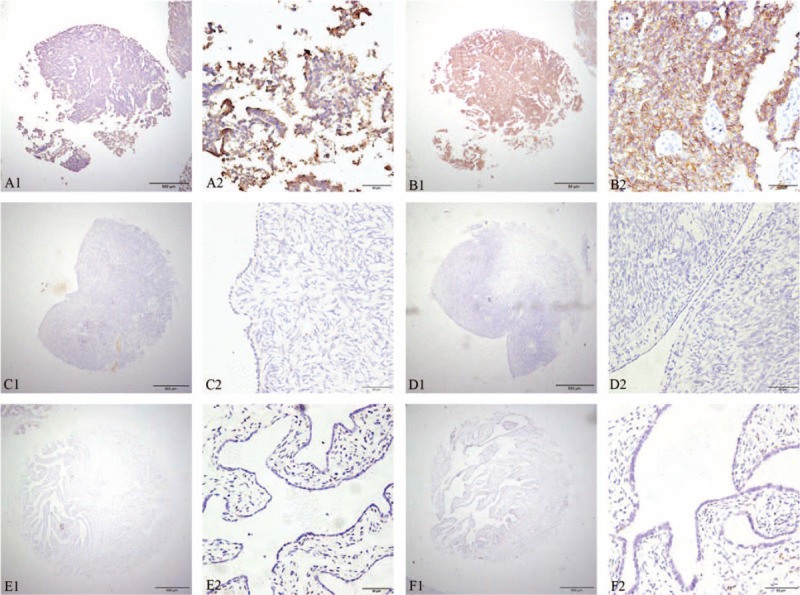
Representation of KIF2A and HER2-neu protein expression in EOC tissue and normal ovarian, fallopian tube tissue. Samples immunostained for KIF2A (a1, a2) show cytoplasmic positivity, as well as different (membrane) subcellular localization for HER2-neu (b1, b2). EOC cells with high KIF2A (a1, a2) and HER2-neu (b1, b2) protein expression; normal ovarian epithelial cells with negative KIF2A (c1, c2) and HER2-neu (d1, d2) protein expression; and normal fallopian tube epithelial cells with negative KIF2A (e1, e2) and HER2-neu (f1, f2) protein expression. Original magnification ×40 in a1, b1, c1, d1, e1, f1 (scale bars 500 μm); ×400 in a2, b2, c2, d2, e2, f2 (scale bars 50 μm). EOC = epithelial ovarian cancer, HER2-neu = human epidermal growth factor receptor 2, KIF2A = Kinesin family member 2A.

IHC analysis was performed to detect KIF2A and HER2-neu in EOC tissue specimens using TMA. Positive staining of KIF2A was predominantly localized in the cytoplasm of EOC cells while HER2-neu was localized in the cytomembrane (Figure 2). Overexpression of KIF2A was observed in 71.17% (79/111) of EOC tumors, 16.67% (4/24) of normal ovarian samples, and 20.83% (5/24) of normal fallopian samples (*P* < 0.01). Overexpression of HER2-neu was observed in 31.53% (35/111) of EOC tumors, 8.33% (2/24) of normal ovarian samples, and 12.50% (3/24) of normal fallopian samples (*P* < 0.01). Chi-squared test analysis indicated that both KIF2A and HER2-neu expression were significantly upregulated in EOC samples, when compared with its general negative or low expression in normal ovarian and fallopian tube tissues (*P* < 0.01; Table 1). Interestingly, high KIF2A and HER2-neu coexpression (KIF2A^H^/HER2-neu^H^) were only detected in EOC patients, and none of normal ovarian or fallopian tissues had KIF2A^H^/HER2-neu^H^ staining, although not all ovarian carcinoma show coexpression of KIF2A and HER2-neu.

**TABLE 1:**
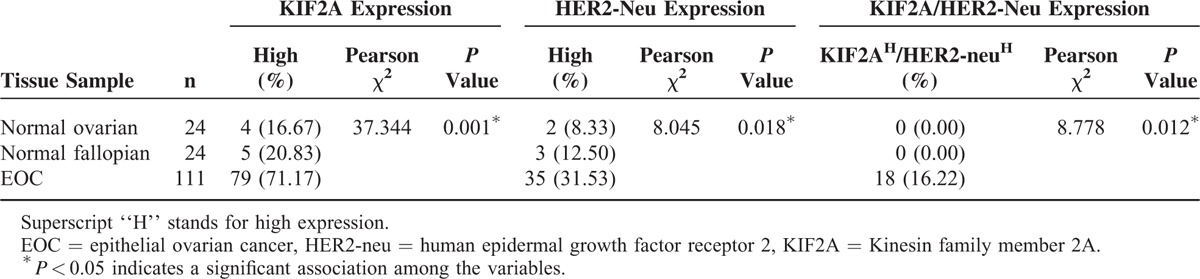
KIF2A and HER2-Neu Immunohistochemical Staining in Normal Ovarian Epithelial Cells, Normal Fallopian Tube Epithelial Cells, and EOC Cells

### Association Between KIF2A and HER2-Neu Expression and Clinicopathological Parameters of EOC

To clarify the potential prognostic roles of KIF2A alone or combined with HER2-neu in EOC development and progression, we evaluated the association of KIF2A and HER2-neu expression with various clinical features of EOC. Associations of KIF2A expression and clinicopathological factors are summarized in Table 2. While stratifying clinical characteristics by KIF2A expression (high vs low), we observed that high KIF2A (KIF2A^H^) protein expression was significantly associated with FIGO stages (*P* = 0.002), ascites cell (*P* = 0.029), metastasis (*P* = 0.027), and lymph nodes (*P* = 0.047; Table 2). However, there was no significant association observed between KIF2A expression and patients’ age, tumor grade, histological type, tumor single or double, level of CA-125, or HER2-neu expression level in the present study (Table 2). Moreover, HER2-neu expression alone showed a positive association with FIGO stages (*P* = 0.001), metastasis (*P* = 0.048), and level of CA-125 (*P* = 0.017). The trends toward its associations with remaining clinical characteristics did not reach statistical significance (Table 2). To test whether high HER2-neu expression (HER2-neu^H^) coupled with KIF2A^H^ in the ovarian epithelium could identify a subgroup patients with a poor outcome, we further compared prognosis among patients with KIF2A^H^/HER2-neu^H^ and non-KIF2A^H^/HER2-neu^H^ (KIF2A^L^/HER2-neu^L^, KIF2A^H^/HER2-neu^L^, and KIF2A^L^/HER2-neu^H^ phenotypes; “H” stands for high expression; “L” stands for low or no expression). We found that simultaneously high KIF2A and HER2-neu expression were significantly related to FIGO stages (*P* < 0.001), metastasis (*P* = 0.030), tumor type (*P* = 0.014), and level of CA-125 (*P* = 0.033; Table 2).

**TABLE 2:**
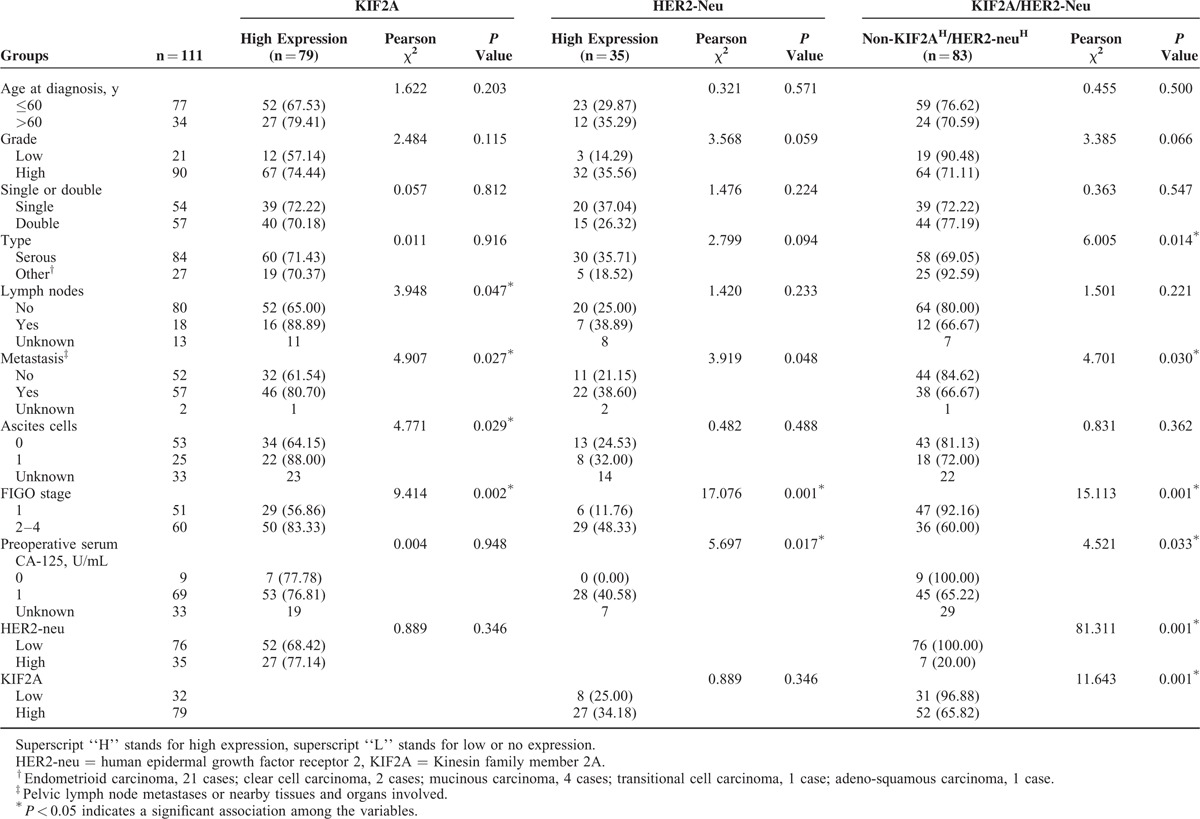
Patient Clinicopathological Characteristics According to KIF2A/HER2-Neu Scores

### High KIF2A Expression Alone or Combined With High HER2-Neu Expression Predicts Poor Prognosis

**FIGURE 3:**
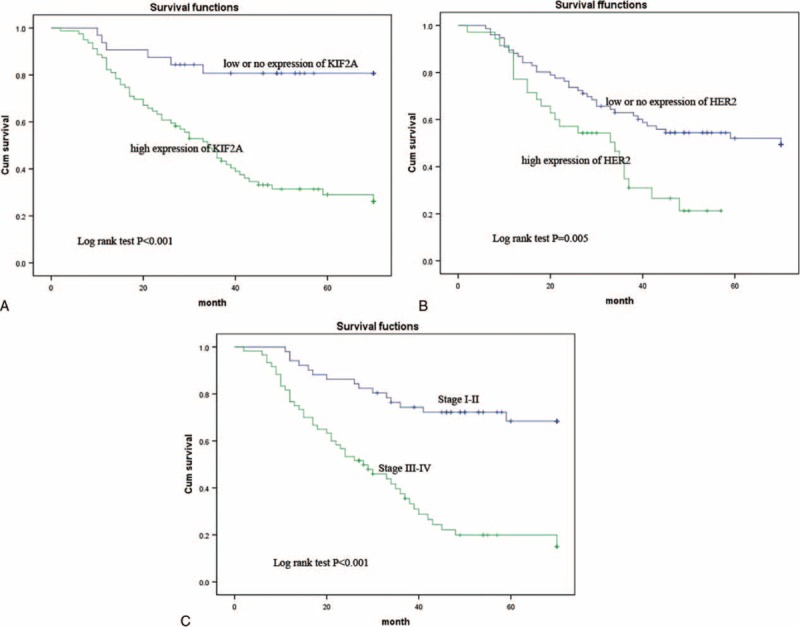
Kaplan–Meier plots using the log-rank survival test. (A) Overall survival (OS) rate in patients with high KIF2A expression was significantly lower than in patients with no or low KIF2A expression. (B) OS rate in patients with high HER2-neu^H^ expression was significantly lower than in patients with no or low HER2-neu^H^ expression. (C) OS was significantly lower in patients with advanced FIGO tumors than in those with lower FIGO tumors. HER2-neu = human epidermal growth factor receptor 2, KIF2A = Kinesin family member 2A.

We also determined prognostic factors in EOC patients using both univariate and multivariate analyses. In the univariate survival analysis, a number of variables were associated with OS, including high-level of KIF2A expression (hazard ratio [HR] = 0.200, 95% confidence interval [CI] 0.086–0.466; *P* < 0.001), high-level of HER2-neu expression (HR = 0.476, 95% CI 0.282–0.803; *P* = 0.005), high KIF2A and HER2-neu (KIF2A^H^/HER2-neu^H^) coexpression (HR = 0.426, 95% CI 0.248–0.733; *P* = 0.002), patient age (HR = 0.476, 95% CI 0.281–0.809; *P* = 0.006), FIGO stage (HR = 0.230, 95% CI 0.127–0.417; *P* < 0.001; Table 3). Thereafter, multivariate analysis was performed using the Cox proportional hazards model for all the significant variables identified in the univariate analysis. In the multivariate Cox regression model, the results demonstrated that KIF2A^H^ (HR = 0.437, 95% CI 0.241–0.795; *P* = 0.007), HER2-neu^H^ (HR = 0.522, 95% CI 0.307–0.890; *P* = 0.017), and FIGO stage (HR = 0.287, 95% CI 0.151–0.545; *P* < 0.001) were unfavorable prognostic factors, independent of other clinicopathological factors (Table 3; Figure 3).

**TABLE 3:**
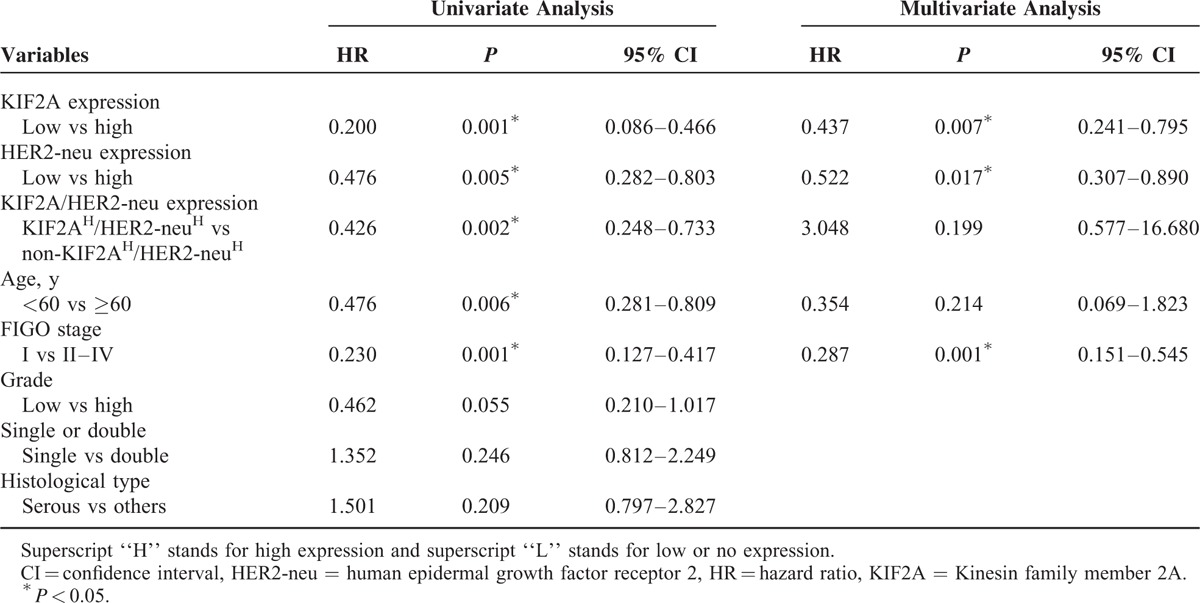
Univariate and Multivariate Cox Proportional Hazard Models of Overall Survival

## DISCUSSION

KIF2A is a member of Kinesin-13 family and involved in cell migration and cell signaling. It is reported that KIF2A has been investigated in a variety of cancers,^14,18,19^ including breast tumor, oral squamous carcinoma, and colorectal cancer. And these findings of KIF2A frequently expression can provide additional prognostic information of many other types of tumors. However, to date, there is no published study examining the potential effect of KIF2A on the survival of patients with EOC. HER2-neu is implicated in the development of many cancers. Both of these 2 proteins are upstream inducer of PI3K/AKT signaling pathway that plays an important role in the regulation of many cellular events including proliferation, survival, and invasion. To better understand the potential roles of KIF2A, we hypothesized that aberrant KIF2A and HER2-neu expression might be associated with aggressive behavior of EOC. We determined the association of KIF2A and HER2-neu expression levels, or a combination of both protein markers with OS in patients with EOC. In this study, we found that the expression level of mitotic centromere-associated Kinesin-13 protein KIF2A was elevated in EOC. The increase in KIF2A expression was associated with a decreased survival time in EOC patient, indicating that KIF2A might be a potential prognosis biomarker for EOC. We also demonstrated that patients with advanced FIGO stage disease and metastasis had more frequent high KIF2A expression in contrast those in the early stage of the disease. These findings suggest that high KIF2A expression may promote tumor progression and metastasis and that KIF2A may be used as a potential biomarker to identify a subgroup with more aggressive phenotype of EOC.

There are 3 distinct genes that encode the Kinesin-13 family members in the human genome, including *KIF2A* (chromosome 5q12), *KIF2B* (chromosome 17q22), and *MCAK*/*KIF2C* (chromosome 1p34). These protein products of these genes participate in intracellular transport, cell division, and bipolar spindle assembly during spindle formation.^20–22^ While cancer cells undergo continuous cell division, incessant chromosomal missegregation may cause the chromosome to lose stability during mitosis.^23,24^ The KIFs are involved in spindle orientation and chromosomal movements during mitosis and cytoskeletal reorganization.^25–27^ It has been shown that KIF2A, essential for bipolar spindle assembly and chromosome movement,^28^ is able to depolymerize microtubule in vitro.^29,30^ KIF2A localizes to spindle poles in human cells. Cells lacking KIF2A fail to form bipolar spindle, but form monopolar in mitosis,^31^ leading to arrest of cell cycle progression. Monopolar spindles in the cells could cause the gain or loss of chromosomes in daughter cells.^28^ Any “error” in this process may cause chromosome missegregation, leading to substantial alteration in cell proliferation and migration.

Previous studies on KIF2A demonstrated that KIF2A silencing induces apoptosis of human oral squamous cell carcinoma Tca-8113 cell lines.^7^ Moreover, several lines of evidence showed that KIF2A is an upstream inducer of the PI3K/AKT pathway, and therefore, it is meaningful to inhibit PI3K/AKT by KIF2A silencing since the over activation of PI3K/AKT is a frequent event in various human cancer.^32–34^ The PI3K/AKT pathway plays a key role in regulating multiple important cellular activities including proliferation, survival, and differentiation.^35^ PI3K/AKT signaling pathway has been extensively investigated as a target for cancer therapy, due to its functions in regulating cell cycle control, driving carcinogenesis, and imparting chemoresistance to anticancer treatment.^36^ A number of candidate drugs targeting this pathway have been discovered and entered clinical trials, such as inhibitors of PI3K, epidermal growth factor receptor (EGFR), platelet-derived growth factor receptor (PDGF-R), and mammalian target of rapamycin (mTOR), as well as monoclonal HER2-neu antibody.

*HER2-neu*, the *ERBB2* proto-oncogene encodes a transmembrane protein tyrosine kinase receptor, which are involved in the occurrence and development of many types of cancers, including ovarian cancer.^37^ Dysregulated HER2-neu signaling in EOC results from either gene amplification or overexpression, which leads to faster cell growth, DNA damage, and increased colony formation.^12^ The PI3K/AKT pathway is a major downstream signaling pathway of HER2-neu. Studies have shown that the addition of PI3K/AKT inhibitors to treatments for patients with HER2-neu-positive tumors and PI3K pathway activation may increase their response to HER2-neu blockade and improve clinical outcomes.^9^ Activation of the PI3K/AKT pathway has been implicated in resistance to anticancer therapies, including chemotherapy, endocrine therapy, and HER2-neu-targeted therapy, and blocking PI3K/AKT pathway signaling may be able to enhance efficacy of these therapies.^38^

In this study, the results showed that both positive KIF2A and HER2-neu staining was significantly related to FIGO stage, though we did not detect correlation between KIF2A and HER2-neu expression. The level of KIF2A expression was strongly associated with an increased risk of metastasis and poor overall survival outcomes. These data indicated that increased KIF2A expression correlated with invasive behavior and metastatic processes of EOC. Both univariate and multivariate analyses demonstrated that KIF2A and HER2-neu expression appeared to be an independent prognostic factor for OS in patients with EOC.

These 2 molecular biomarkers (KIF2A and HER2-neu) may help to identify patients who may benefit from close postsurgery monitoring and design optimal individualized treatment plans. A previous successful application of biomarkers in cancer treatments was found in which patients with tumors that had both PIK3CA mutation and PTGS2 expression respond best to aspirin therapy.^39^ Additional research would be required to confirm our findings.

Despite some limitations, such as the small number of patients and relatively short follow-up time, our results provide the first evidence that KIF2A might be used as a novel biomarker to identify EOC patients with “poor diagnosis” after surgery and ameliorate personalized medicine. Our analysis showed that level of KIF2A and HER2-neu is elevated in EOC patients. Furthermore, high expression of KIF2A and HER2-neu were associated with a less favorable long-term outcome. However, large, well-designed, randomized controlled studies are warranted to validate the potential clinical value of KIF2A, either alone or in conjunction with HER2-neu in EOC.

In conclusion, this study may advance our understanding of the role of KIF2A in the progression and development of EOC. Further studies are needed to investigate related signaling pathways such as PI3K/AKT pathway and potential mechanisms underlying the oncogenic impact of KIF2A overexpression in EOC, which may help to fully understand and exploit the prognostic and therapeutic value of KIF2A.
